# Circular mRNA against CleanCap linear mRNA vectors: comprehensive comparison, expression, active and passive immunization

**DOI:** 10.3389/fimmu.2026.1734751

**Published:** 2026-04-24

**Authors:** Vladimir M. Vakhtinskii, Irina L. Tutykhina, Alina S. Dzharullaeva, Daria M. Grousova, Ilya D. Zorkov, Anna A. Ilyukhina, Dmitrii A. Reshetnikov, Valentin V. Azizyan, Artem A. Derkaev, Evgeniia N. Bykonia, Evgeny V. Usachev, Denis A. Kleymenov, Vladimir A. Gushchin, Inna V. Shuliakova, Dmitry V. Shcheblyakov, Maxim M. Shmarov, Denis Yu. Logunov, Alexander L. Gintsburg

**Affiliations:** 1 Federal State Budget Institution “National Research Center for Epidemiology and Microbiology Named after the Honorary Academician N.F. Gamaleya”, The Ministry of Health of the Russian Federation, Moscow, Russia; 2 Sechenov First Moscow State Medical University, Moscow, Russia; 3 Department of Virology, Lomonosov Moscow State University, Moscow, Russia

**Keywords:** ARCA, circular mRNA, circular mRNA-vaccine, CleanCap, CVB3 IRES, HRVB6 IRES, SARS-CoV2 vaccine, universal vector platform

## Abstract

**Introduction:**

The mRNA platform has revolutionised vaccine technology by offering a universal, rapid, and easily scalable production process. Two main types of mRNA vectors exist—linear (cap‑dependent) and circular (cap‑independent)—each with distinct advantages. Although both vector types are continuously being improved, a comprehensive comparative analysis of the most efficient existing vectors of each type has been lacking.

**Methods:**

We compared the expression efficiency, protective activity, and therapeutic activity of circular and linear mRNA vectors. Linear vectors were tested in different configurations: containing either N1‑methylpseudouridine or uridine, and capped with either ARCA (m^7^G(5′)ppp(5′)G) or CleanCap (m^7^G(5′)ppp(5′)m^2^G). Circular vectors contained either the commonly used IRES of coxsackievirus B3 or a new IRES of human rhinovirus B6. Expression levels were evaluated using a luciferase reporter assay and target protein expression. Protective activity was assessed through both active immunization (immunogenicity and subsequent SARS-Cov2 challenge) and passive immunization (recombinant antibody production and toxin challenge).

**Results:**

Preliminary luciferase assays showed that modified linear vectors achieved significantly higher expression levels both *in vitro* and *in vivo*. A similar, though less pronounced, difference was observed for target protein expression. In active immunization studies, immunogenicity and protective activity of circular vector were equal to those of linear. While for passive immunization model, linear vectors conferred significantly better protection than circular vectors, that correlates directly with their higher protein expression levels observed *in vivo*.

**Discussion:**

Despite the markedly higher expression levels observed with modified linear vectors both in vitro and in vivo we didn`t observe the superiority in immunogenicity or protection in active immunization experiment. At the same time for passive immunization, requiring high expression levels of target protein linear vectors seems to be better choice.

## 1 Introduction

mRNA-based vaccines have already demonstrated a high level of safety and protective activity during SARS-CoV-2 pandemic (1).

In comparison to traditional vaccines, the mRNA platform offers a number of benefits. Among these are a standardized technological process, independent of the target pathogen, which facilitates large-scale production and simplifies regulatory approval (2).

There are two main types of mRNA vectors: linear (cap-dependant) and circular (cap independent). Both have their advantages and disadvantages. Linear vectors are better studied and more used in vaccine production. One of the best examples is a widely used vaccine against SARS-CoV-2 which is based on cap-dependent mRNA technology (1, 3, 4).

Circular mRNA vectors are relatively new, nevertheless, in recent years a number of vaccines based on this technology have showed their immunogenicity against various viral pathogens, including the Zika virus, Influenza viruses, Rabies-virus, and Monkeypox virus. Finally, circular mRNA was used as a platform for cancer vaccine (5–9).

The efficacy of circular mRNA is mainly determined by the Internal Ribosome Entering Site (IRES). There is a great diversity of known IRESes, but most of them are poorly studied and not used routinely. A set of frequently used IRESes, originates from human rhinoviruses A and B (HRV-A1. HRV-B3), coxsackievirus B3 (CVB3), encephalomyocarditis virus (EMCV), cricket paralysis virus (CrPV), poliovirus 1 (PV1) and hepatitis C virus (HCV). Among these the CVB3 IRES is a major one (10).

Expression levels of linear mRNA-vectors are based on the cap type and chosen 5` and 3` untranslated regions (UTRs). Cap is a structure at 5’ end of mRNA of eukaryotes. There are two main types of natural cap structures cap0 – 7-methylguanosine, linked by 5’-5’-triphosphate bridge (m^7^GpppNp) and cap1 – which contains additional methyl group in the first nucleotide (m^7^GpppNm^2^’Op).

There is an *in vitro* capping technology, providing single-step capping with cap0 type and an optional second stage for the enzymatic formation of cap1. Nevertheless, this approach is rather expensive. Another option is a use of cap analogues, such as Anti-Reverse Cap Analogue (ARCA). This technology offers a single-step co-transcriptional capping; however, its efficiency is about 80%. By now, the most effective and widely used capping reagent is m^7^G(5′)ppp(5′)m^2^G also known as CleanCap AG. As an example, it was used for production of BNT162b2 vaccine against SARS-CoV2.

Important role belongs to UTRs, certain researches investigate natural UTRs, others screen the libraries of artificial UTRs. The most widely used and effective UTRs to the date are UTRs of alfa-globin, that were applied in BNT162b2 SARS-Cov2 vaccine (11–14).

A number of studies are devoted to the comparison of luciferase activity of circular and linear vectors, but fewer studies have provided immunogenicity and protective activity data. Among them are the work of Wan, J et all, and the work of Qu L. et al. Wan, J et al. compared the immunogenicity of linear and circular vectors on the model of surface glycoprotein G of Rabies virus. And Qu L. et al. studied used as a model antigen RBD-domain of S-glycoprotein of SARS-Cov2 (7, 15).

Interesting, that both Wan, J et all and Qu L. et al. used linear vectors, capped with first-generation cap analogues (m^7^G(5′)ppp(5′)G, while the approved linear mRNA-vaccines use more effective third-generation CleanCap reagent (m^7^G(5′)ppp(5′) m^2^G) (15).

That is why the aim of our study was to compare immunogenicity and protective activity of different linear mRNA capped with various cap types against circular mRNA vector containing different IRESes. As an etalon of UTRs of linear mRNA vector, we chose the UTRs of alfa-globin, as they are considered among the most efficient and safe.

Recent research claims that the IRESes of some Rhinoviruses are more effective than CVB3. Therefore, in our work, in addition to “popular” IRES CVB3 we also used the yet-not studied IRES of Rhinovirus B6 (HRV B6) (16).

## 2 Results

### 2.1 Linear and circular mRNA-vectors

**Figure 1:**
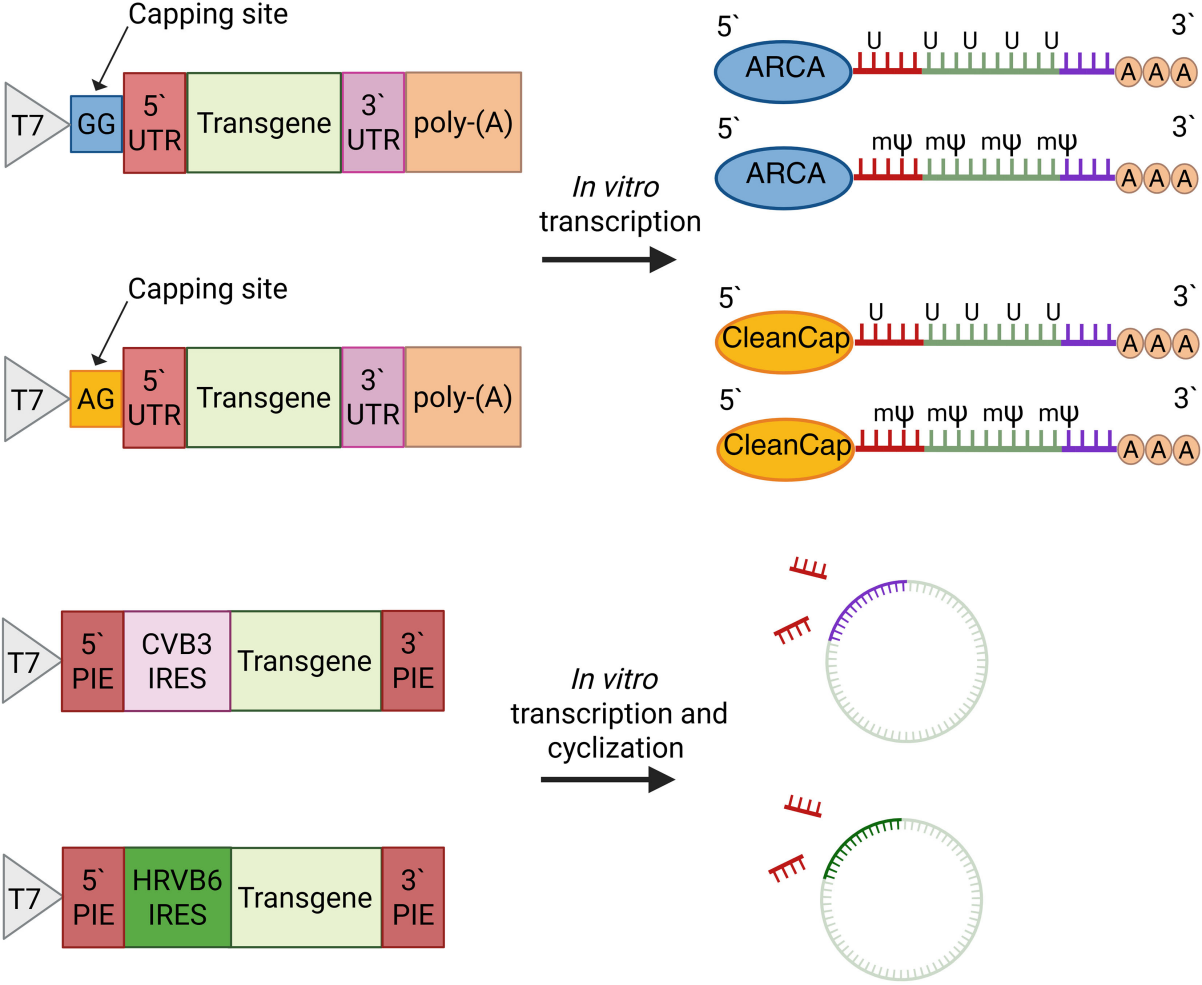
Schematic process of mRNA-vectors production. Linear vectors contained either uridine or N1-methylpseudouridine and were capped with ARCA reagent or CleanCap AG reagents. Circular vectors include either CVB3 or HRVB6 IRES and did not contain N1-methylpseudouridine.

The synthesis scheme of linear and circular vectors is shown in **Figure 1**. All the templates contain T7 promoter, which is recognized by T7 RNA-polymerase.

Two linear templates have 5`and 3` untranslated regions (5`UTR and 3`UTR) from alfa-globin, the gene of interest, and poly-(A) sequence, consisting of 30 and 70 residues separated by a 10-nucleotide “island”. The difference between the two linear vectors lies in the two nucleotides after the T7 promoter, which define the capping system (AG was used for CleanCap AG reagent, while GG was used for ARCA-capping). Both linear vectors were made with uridine or with its substitution with N1-methylpseudouridine. Purity of obtained vectors was evaluated by 260/280 absorbance ratio, stability of RNA was confirmed via agarose gel-electrophoresis (**Supplementary Figures 1**–**7**).

Circular vectors have special permuted intron-exons (PIE) required for mRNA cyclization at the 5` and 3` ends of the mRNA IRES and the gene of interest. The distinction between them is in the IRES used: CVB3 or HRVB6.

Linear vectors were produced using a single-step procedure of mRNA-capping, which occurs co-transcriptionally, followed by XRN-1 and RppH treatment. In contrast, circular vectors required a second step of cyclization followed by the treatment with RNase R to remove introns and linear precursors.

### 2.2 *In vitro* luciferase activity

**Figure 2:**
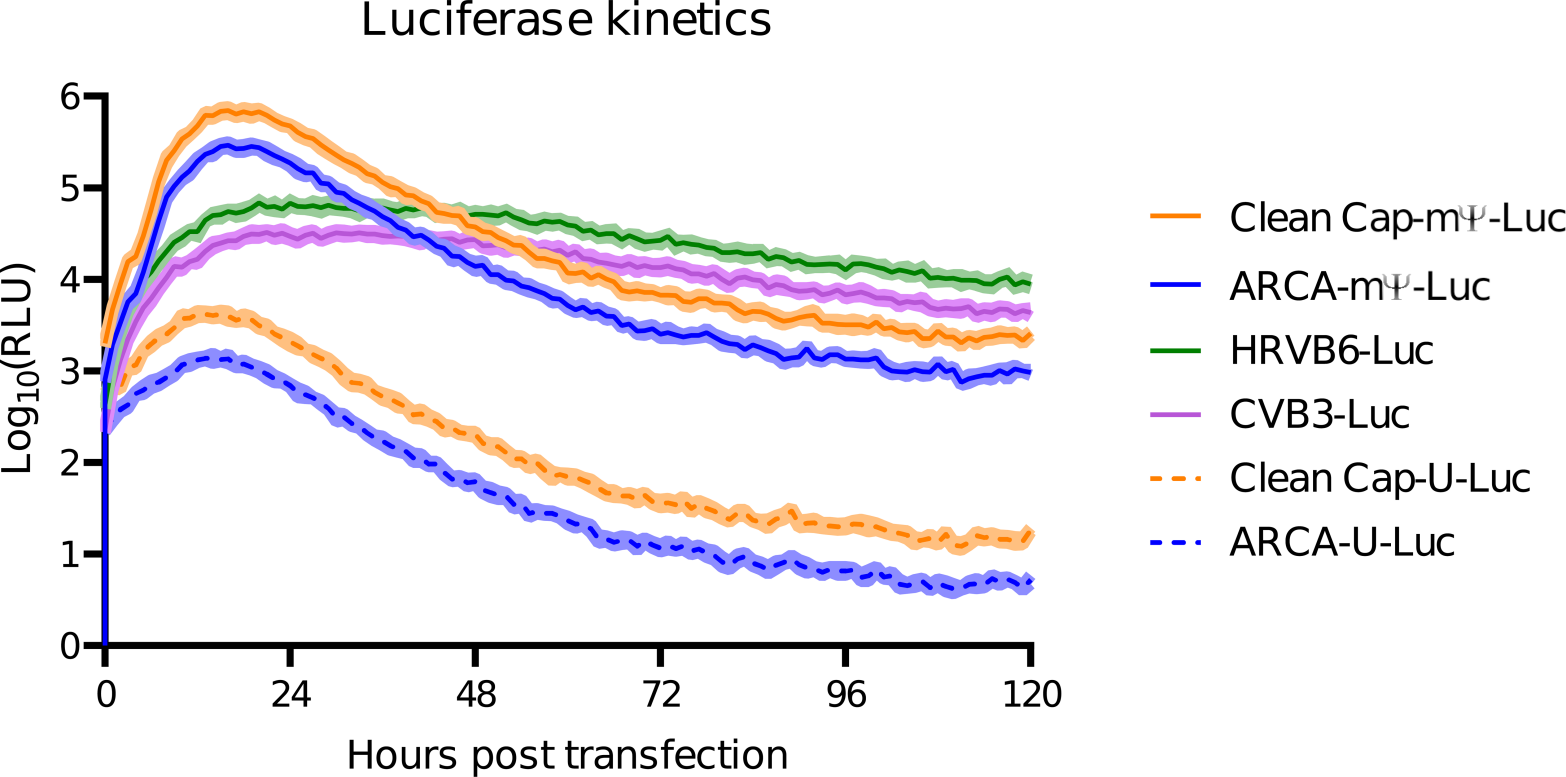
*In vitro* kinetics of luciferase activity in HEK-293 cell line. The mean is indicated as line, CI 95% interval is indicated as shaded regions. (The experiment was performed three times at different cell passages; for each experiment all the mRNA-vectors were freshly prepared.

The first step of our experiment was to compare the *in vitro* expression of different constructs using a firefly luciferase assay. HEK-293 cells were transfected with different mRNA-vectors. The 120-hour-long kinetic curves of luciferase activity are presented in **Figure 2**. Values of luciferase activity at selected time-points are presented in **Table 1**.

**Table 1:** *In vitro* luciferase activity at selected time-points (mean with CI 95%).

mRNA-vector	10 hours	24 hours	48 hours	72 hours	96 hours	120 hours
CleanCap mΨ	340 144.3 ± 29 534.5	472 710.2 ± 17 678.9	37 323.7 ± 3 602.7	6 732.3 ± 251.8	3 193.7 ± 326.2	2 569.3 ± 157.6
ARCA-mΨ	130 494.3 ± 10 832.9	185 766.8 ± 16 258.3	138 76.1 ± 1 698.3	2 517.6 ± 258.9	1 345.9 ± 63.8	964.8 ± 67.7
HRVB6	28 538.7 ± 2 605.1	68 002.1 ± 5 582.4	51 310.4 ± 4 404.4	26 724.8 ± 2 696.7	12 672.5 ± 953.7	8 849.6 ± 872.7
CVB3	13 530.4 ± 1 245.8	29 550.2 ± 2 899.5	27 071.6 ± 1 804.4	13 430.3 ± 1 405.7	6 752.5 ± 680.3	4 328.6 ± 490.9
CleanCap-U	3 735.7 ± 401.7	2 075.3 ± 244.9	202.9 ± 13.5	35.6 ± 3.7	20 ± 1.7	18.3 ± 1
ARCA-U	1 166.8 ± 89.1	694.2 ± 39	62.0 ± 5.2	11.7 ± 1.1	6.5 ± 0.6	5.3 ± 0.6

As shown, the peak of luciferase activity for linear vectors occurs between 16 and 24 hours, while for “circular” vectors the maximum is observed at 24 hours.

Linear vectors containing N1-methylpseudouridine demonstrated significantly higher luciferase activity compared to circular vectors. At the same time linear non-modified vectors exhibit low luciferase activity and a more dramatic decline over the time. By 24 hours the activity of non-modified linear vectors had decreased more than that of modified vectors.

The highest luciferase activity was observed for the linear vector capped with CleanCap reagent and modified with N1-methylpseudouridine at the 24- and 10-hour time-points: 472 710.2 ± 17 678.9 and 340 144.3 ± 29 534.5 respectively. The second highest activity was observed in a linear modified vector but capped with ARCA reagent, at the same time points: 185 766.8 ± 16 258.3 and 130 494.3 ± 10 832.9 respectively.

The circular vector with HRVB6 IRES ranked third with luciferase activity 68 002.1 ± 5 582.4 at 24-hour time point. The circular vector with CVB3 IRES was fourth with luciferase activity 29 550.2 ± 2 899.5 at 24-hour time point. Linear non-modified vectors showed poor results, not exceeding 4000.

Further *in vitro* kinetics studies were limited by a number of factors. Firstly, CLARIOstar plate reader (BMG Labtech) lacked the required humidity causing the outer wells of the plate to dry slow. Secondly, after five days without reseeding, the cells entered the stationary phase and began detaching from the bottom of the wells. Nevertheless, we anticipate that the decline in luciferase activity of the circular mRNA vectors will be less dramatic than that of the linear vectors over the next few days.

Regarding these results of the *in vitro* luciferase activity, we excluded both linear mRNA with uridine from further *in vivo* experiments as they were the least effective in human considerations.

### 2.3 *In vivo* luciferase activity

**Figure 3:**
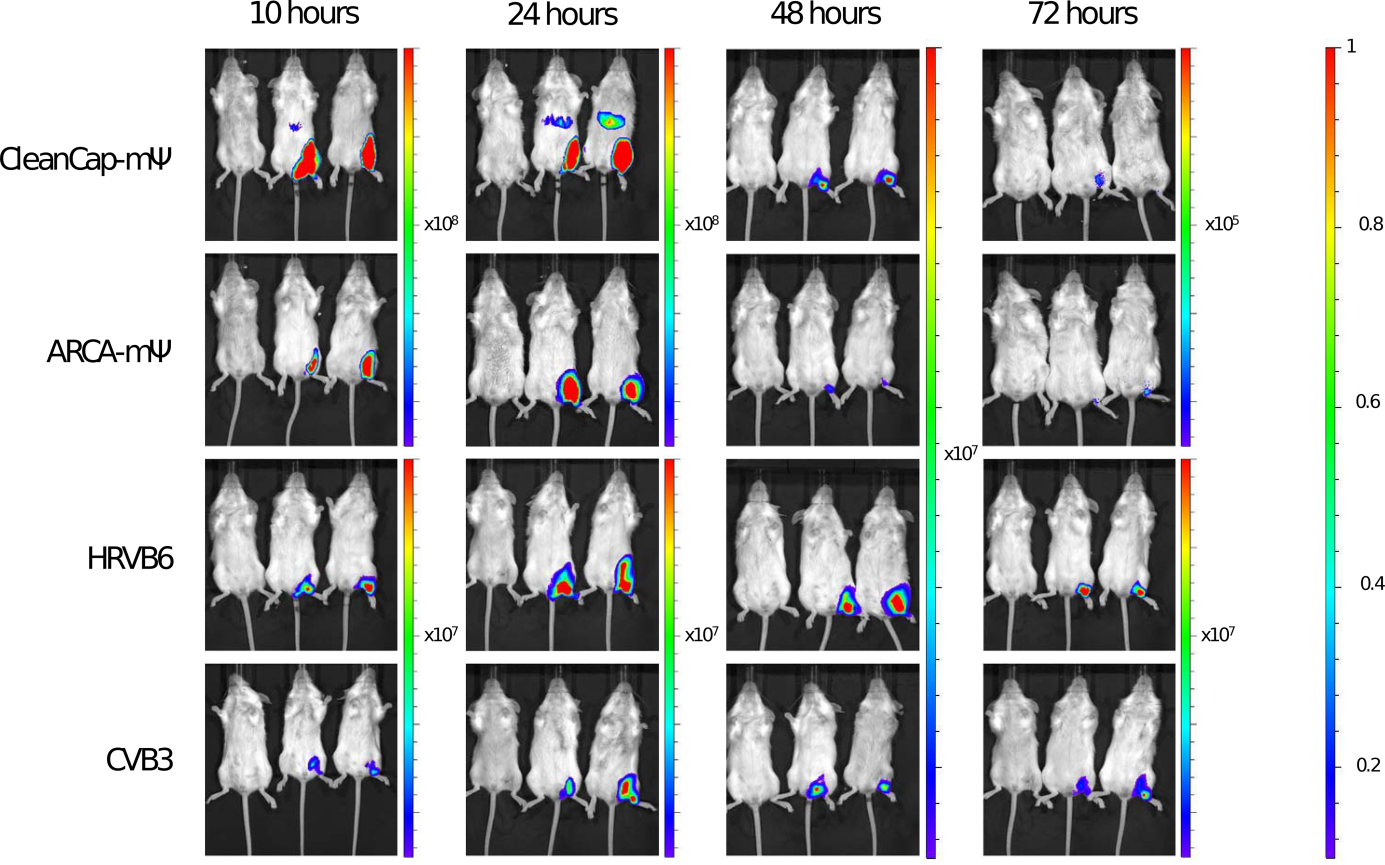
*In vivo* luciferase activity in BALB/c mice at various time-points.

BALB/c female mice were injected intramuscularly with lipid nanoparticles (LNPs), containing different luciferase-coding vectors. The dose of mRNA was 10 µg per mouse. At various time points, luciferase activity was measured using an IVIS Lumina II (Life sciences). The visualised results are shown in **Figure 3** and quantitative data is presented in **Table 2**.

**Table 2:** Total calculated *in vivo* luciferase activity, [p/s].

Vector	mice	Total flux [p/s]			
		10 hours	24 hours	48 hours	72 hours
CleanCap mΨ	1	6.42x10^9^	7.62x10^9^	5.25 x10^7^	2.50 x10^5^
	2	4.19 x 10^9^	5.70X10^9^	4.22 x10^7^	1.31 x10^5^
	mock	4.73 x10 ^7^	8.69 x10^7^	1.95 x10^5^	1.07 x10^5^
ARCA mΨ	1	1.73 x10^9^	6.72 x10^9^	8.10 x10^6^	1.09 x10^5^
	2	2.96 x10^9^	3.86 x10^9^	5.80 x10^6^	1.64 x10^5^
	mock	4.42 x10^6^	4.17 x10^7^	1.45 x10^5^	9.93 x10^4^
HRVB6	1	6.57 x10^7^	5.27 x10^8^	1.47 x10^8^	7.67 x10^7^
	2	1.10 x10^8^	5.75 x10^8^	2.28 x10^8^	1.18 x10^8^
	mock	2.53 x10^5^	8.25 x10^5^	3.53 x10^5^	2.69 x10^5^
CVB3	1	2.74 x10^7^	4.25 x10^7^	4.43 x10^7^	1.74 x10^7^
	2	2.61 x10^7^	1.52 x10^8^	2.34 x10^7^	4.09 x10^7^
	mock	2.00 x10^5^	2.79 x10^5^	2.57 x10^5^	1.93 x10^5^

*In vivo* luciferase activity results were consistent with the *in vitro* findings. Mice treated with linear mRNA vector reached near-maximum values at 10 and 24 hours, followed by a dramatic decrease in luciferase activity over the next 24 hours. Circular vectors showed a slower decline in luciferase activity.

As *in vitro*, the highest total luciferase activity *in vivo* was observed for the linear vector capped with CleanCap reagent and modified with N1-methylpseudouridine at the 24-hour time-point. The second highest was the linear modified vector capped with ARCA reagent also at the 24-hour time-point. Circular vectors HRVB6 and CVB3 were ranked third and fourth respectively.

Our next step was to evaluate the expression levels of target protein on the model of the SARS-CoV-2 S glycoprotein. This gene was inserted into each of the vector types. Stability of the S-coding RNA was confirmed via agarose gel-electrophoresis. Purity was evaluated by 260/280 absorbance ratio (**Supplementary Figures 1**-**6**, **8**).

### 2.4 Expression of S-glycoprotein of SARS-Cov2

**Figure 4:**
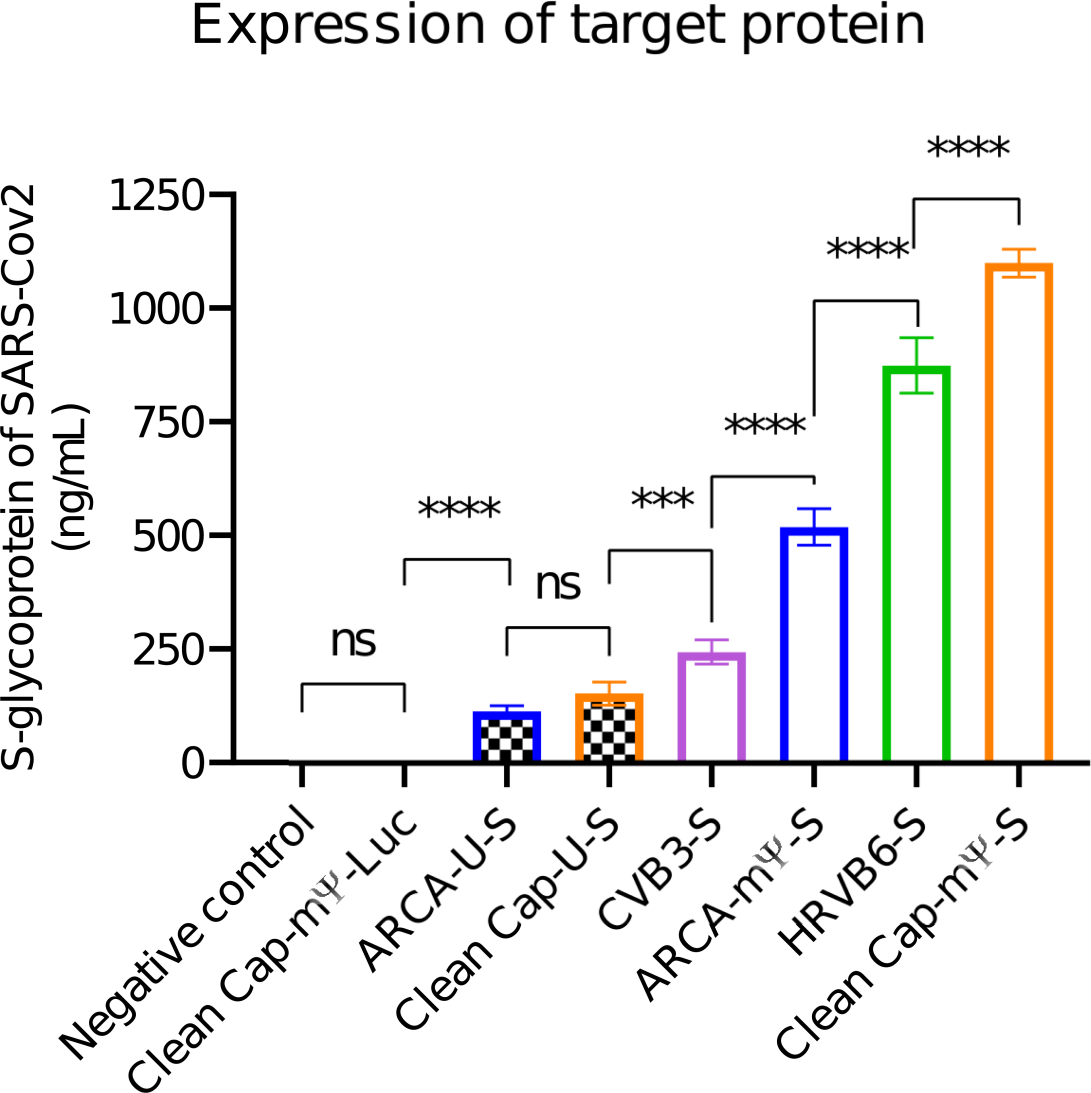
*In vitro* expression of SARS-CoV2 S-glycoprotein in HEK-293 cells 20 hours post-transfection. Data is presented as a mean with 95% CI intervals. P-values were determined by the non-parametric ANOVA (Friedman’s test) with Dunn’s multiple comparison post-test ***p=0.0002; ****p<0.0001, ns = not significant.

HEK-293 cells in a 6-well plate with were transfected with different mRNA vectors. As an additional negative control, we used the most productive luciferase-coding vector (CleanCap-mΨ-Luc). Twenty hours post transfection, cells were washed and lysed. Cell lysate supernatants were used for ELISA against the S-glycoprotein of SARS-CoV2. The results are shown in **Figure 4**, and the mean values with 95% confidence intervals are presented in **Table 3**.

**Table 3:** Expression levels of SARS-CoV2 S-glycoprotein in HEK-293 cells (mean and 95% CI)* ng/mL.

Vector	ARCA-U-S	CleanCap -U-S	CVB3-S	ARCA-mΨ-S	HRVB6-S	CleanCap-mΨ-S
Concentration, ng/mL	114.3 ± 12.5	153.2 ± 26.45	244.7 ± 27.95	519.5 ± 42.1	875.0 ± 63.7	1100 ± 32

* Zero means of luciferase-coding vector and negative control are not shown.

As well as in the luciferase assay the highest expression was observed for the linear vector capped with CleanCap reagent and modified with N1-methylpseudouridine (1100 ± 32). Surprisingly the second-higher expression level belonged to the circular mRNA containing the HRVB6 IRES (875.0 ± 63.7). The linear modified vector capped with the ARCA reagent ranked third with 519.5 ± 42.1 ng/ml. The circular vector with the CVB IRES showed 244.7 ± 27.95 ng/ml. Linear non-modified vectors exhibited poor expression: 153.2 ± 26.45 and 114.3 ± 12.5 for the CleanCap and for the ARCA respectively.

Surprisingly, expression of S-glycoprotein of SARS-Cov2 by circular mRNA-vector, containing IRES HRVB6 was close to linear CleanCap-mΨ-S (875 ng/mL and 1100 ng/mL, respectively). For linear mRNA modified with N1-methylpseudouridine (m1Ψ the difference between CleanCap-S and ARCA-S was approximately 2 times (1100 ng/mL against 519 ng/mL). The difference between the circular mRNA-vector containing CVB3 IRES and the linear CleanCap-mΨ-S decreased from 16-fold in the luciferase assay to 4.5-fold in antigen expression.

We selected the 20-hour time point for the *in vitro* protein-expression experiment with the luciferase assay. By the 20^th^ hour, the linear vectors remain on the plateau, while the circular vectors have just reached it, making this an optimal moment for comparing “maximum” expression levels.

Next, we investigated the immunogenicity of the generated vectors in mice. As during *in vivo* luciferase assay, we excluded linear vectors containing canonical nucleotides in human consideration, because of their instability.

### 2.5 Immunogenicity study

The first step was a single-dose intramuscular immunization of ACE-2 mice with mRNA at a dose of 10 µg/mouse or PBS for the negative control. Each group contained 10 mice.

**Figure 5:**
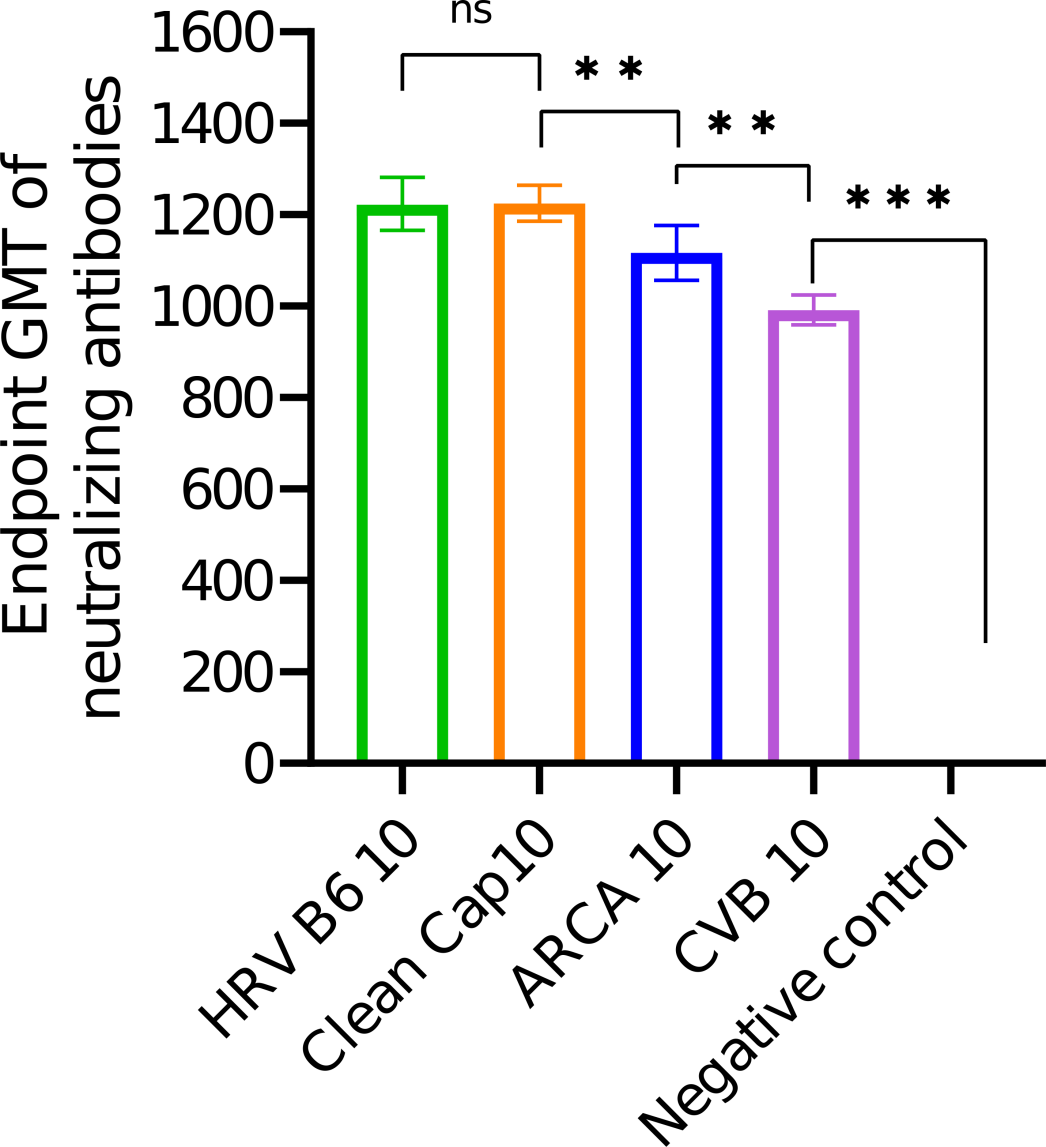
Endpoint GMT of anti-RBD domain neutralizing antibodies in the serum of mice after a single-dose immunization at a dose of 10 µg per mouse. Data is presented as geometric mean with 95% CI intervals. P-values were determined by the Mann–Whitney test **p< 0.0078; ***p<0.0005.

On day 21 post-immunization we measured an antibody titre against the RBD-domain of SARS-CoV-2 (Wuhan) in the serum of the vaccinated animals. Graphical results are shown in **Figure 5** and the endpoint GMT is presented in **Table 4**.

**Table 4:** Endpoint GMT of anti-S-glycoprotein of SARS-CoV2 in serum of mice after a single-dose immunization at a dose of 10 µg per mouse. (Geometric mean and 95% CI)*.

Vector	HRVB6-S	CleanCap-mΨ-S	ARCA-mΨ-S	CVB3-S
Endpoint GMT	1222 ± 83	1224 ± 40	1113 ± 60	990 ± 48

* Zero means of negative control is not shown.

Results of a single-dose immunization showed comparable endpoint GMTs for the linear vector capped with CleanCap reagent and modified with N1-methylpseudouridine and the circular vector with the HRVB6 IRES (1224 ± 40 and 1222 ± 83, respectively). The circular vector with the CVB3 IRES had the lowest endpoint GMT (990 ± 48), while the linear vector capped with the ARCA reagent and modified with N1-methylpseudouridine showed an endpoint GMT 1113 ± 60.

For subsequent work we excluded the two lowest-performing vectors, being a linear mRNA with ARCA and circular mRNA with CVB3 IRES. In the next experiment we used a classical prime-boost immunisation with the reduced doses (3 µg per mouse).

### 2.6 Immunogenicity and protective activity of two “best” vectors

ACE-2 mice were divided into three groups with 30 mice in each. Animals were immunized twice at a dose 3 µg/mouse. The interval between first and second doses was 21 day.

**Figure 6:**
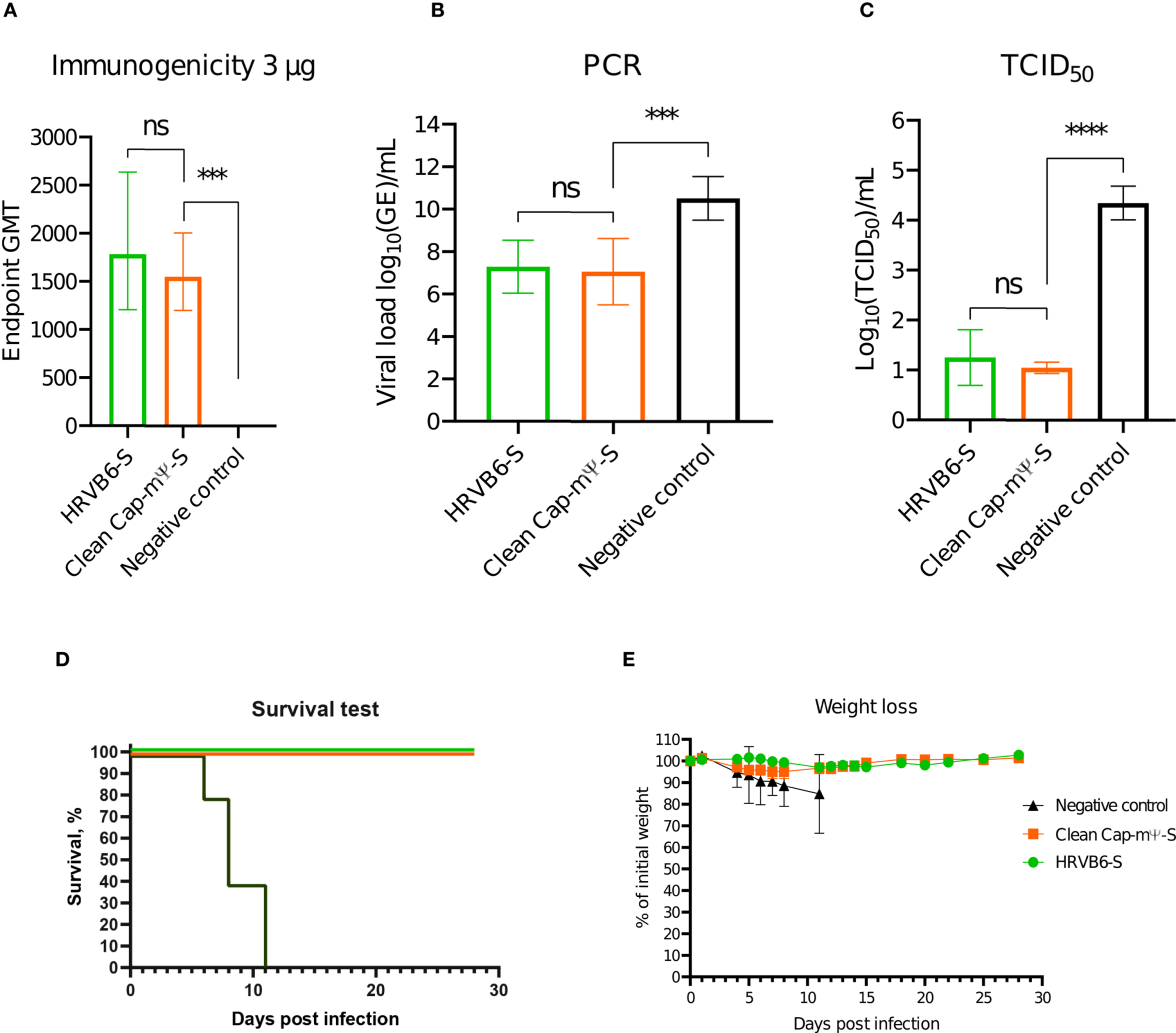
**(A)** Endpoint GMT of anti-RBD domain neutralizing antibodies after prime-boost vaccination at a dose 3 µg. **(B)** Viral load measured by PCR-RT in lungs of infected mice on day 3 post SARS-CoV2 challenge. **(C)** Viral load measured by TCID50 titration in lungs of infected mice on day 3 post SARS-CoV2 challenge. **(D)** Survival after SARS-CoV2 challenge. **(E)** Weight loss after SARS-CoV2 challenge. ns = not significant. ***p=0.0007; ****p<0.0001.

On day 21 after the second immunization, 10 mice from each group were selected for blood collection and subsequent antibody titre measurement. The results are shown in **Figure 6A**.

The remaining mice were challenged with the SARS-CoV2 Wuhan strain at a dose 10^5^ TCID_50_. On the 3^rd^ day post-infection 10 mice from each group were sacrificed. Viral load in the lungs was measured using RT-PCR and TCID_50_ assay on Vero cells. The results are presented in **Figures 6B, C**. The remaining mice were monitored for four weeks with daily weight measurements (**Figures 6D, E**).

Both groups of immunized mice showed equivalent GMTs of anti-RBD neutralizing antibodies, significantly higher than the negative control (1783 ± 715 for the circular vector with HRVB6 IRES and 1552 ± 402 for the linear CleanCap vector).

Viral load measured by RT-PCR also showed a significant decrease in viral load in lungs. Log_10_ of genome copies were 7.283 ± 1.251 for circular vector and 7.063 ± 1.559 for the linear vector compared to 10.52 ± 1.027 in the control group.

TCID_50_ titration also indicated lower viral loads in vaccinated mice. Log_10_(TCID_50_) was 1.25 ± 0.694 for the circular vector and 1.05 ± 0.139 for the linear vector, versus 4.35 ± 0.416 for the control group.

Survival test showed 100% protection from lethal outcome in both linear and circular vector-immunized groups, while all unvaccinated mice died by day 11. The results of daily weigh monitoring indicated that all the mice lost weight during the first 11 days post-infection, after which vaccinated mice began regaining weight. Mice immunized with the linear vector experienced less weight loss, compared to those vaccinated with the circular vector. Nevertheless, by day 26 post-infection all vaccinated groups reached their initial weight.

### 2.7 Application of circular mRNA-vector for passive immunization

To complete a multifaceted comparison, we evaluated the protective efficacy of circular mRNA-vectors expressing recombinant antibody against *C. botulinum* toxin A (BonT/A).

We generated a circular vector, containing IRES HRVB6 coding B11-Fc antibody. The linear vector capped with CleanCap reagent was described previously (17). Stability of obtained RNA was confirmed via agarose gel-electrophoresis. Purity was evaluated by 260/280 absorbance ratio, (**Supplementary Figures 1**-**6**, **9**).

**Figure 7:**
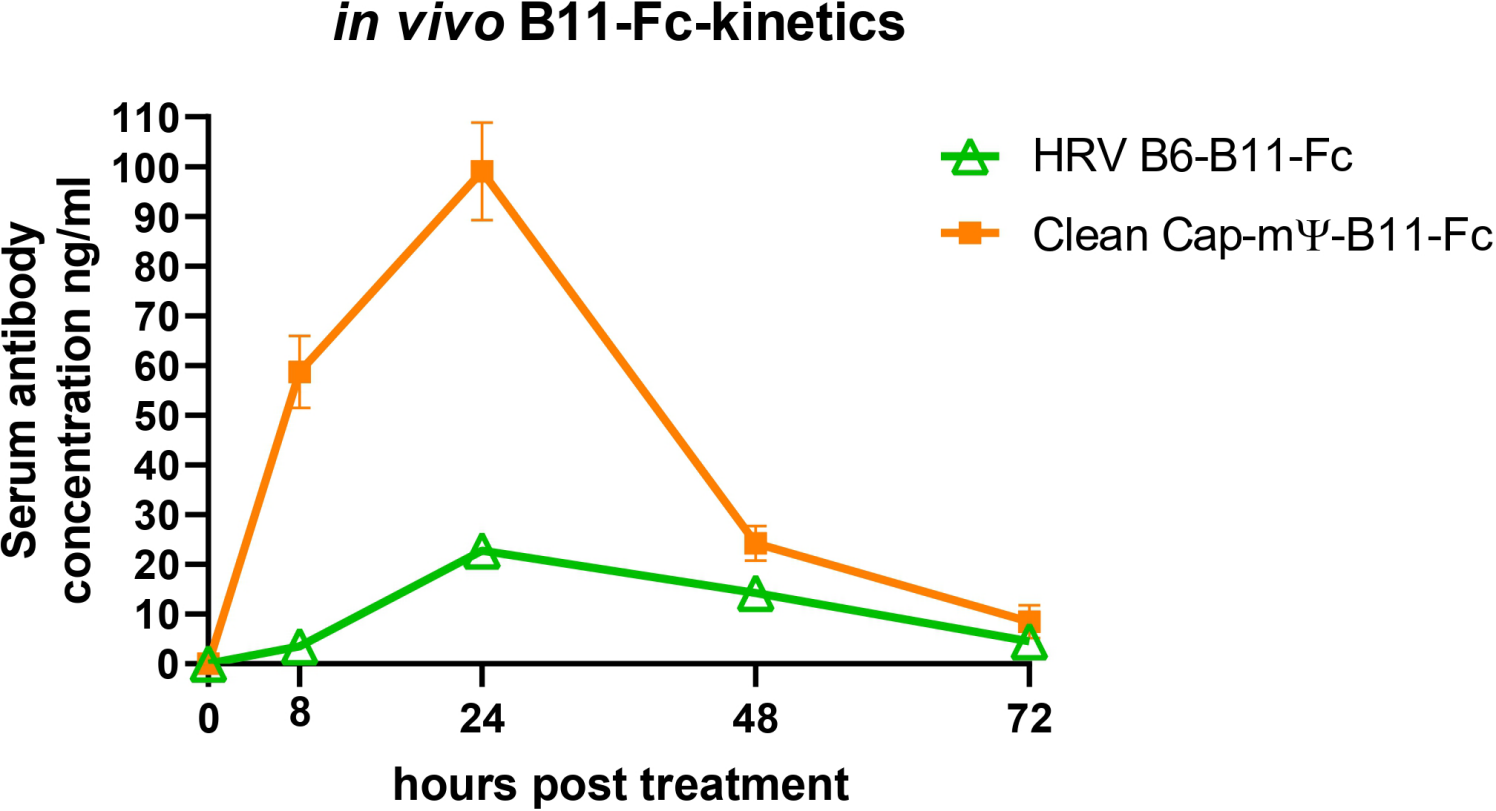
*In vivo* kinetics curve of B11-Fc antibody expression by the linear and the circular mRNA vectors.

Mice were divided into three groups of 12 animals each and were treated with LNPs, containing either a linear or circular vector at a dose of 10 µg per mouse. As a negative control a luciferase-coding linear vector was used. At various timepoints the mice underwent blood collection and the subsequent recombinant antibody titre measurement in serum by ELISA. The results are shown in **Figure 7**.

*In vivo* kinetics of B11-Fc expression by mRNA vectors showed a higher level of expression for the linear vectors at all the time points. Maximum values were 99 ± 9,8 ng/ml for linear vector and 22,75 ± 2,7 ng/ml for the circular vector at the 24-hour time-point.

We then compared the protective activity of the obtained circular vector with the linear one. For this experiment mice were divided into three groups of 16 animals each and were treated with LNPs as in previous experiment. At 4, 16, 24 and 48 hour post-treatment mice were challenged intraperitoneally with 5LD_50_ of BonT/A The survival test results are shown in **Figurse 8A-D**.

**Figure 8:**
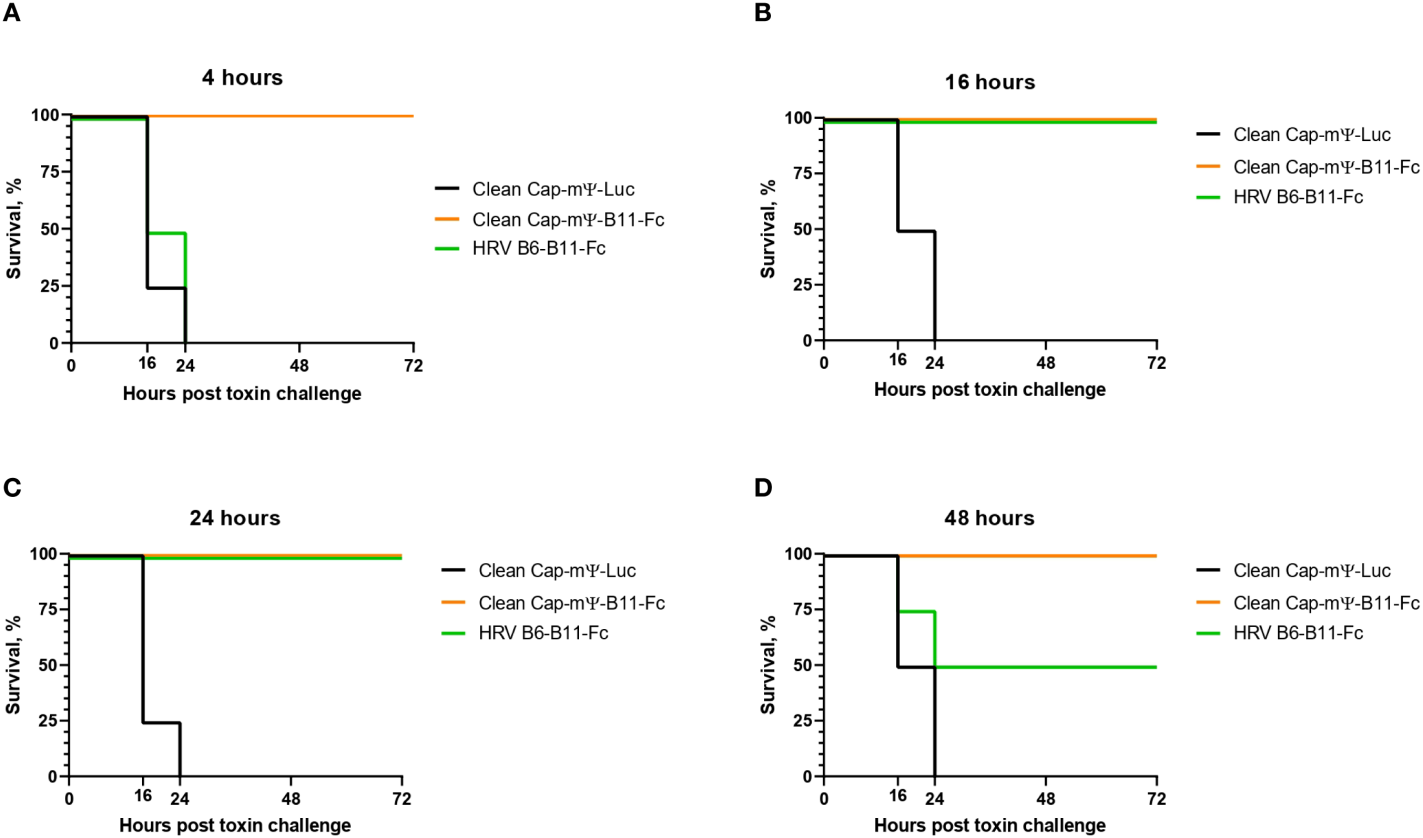
Survival test after 5LD_50_ BonT/A challenge of treated mice. **(A)** mRNA treatment 4 hours before toxin challenge, **(B)** mRNA treatment 16 hours before toxin challenge, **(C)** mRNA treatment 24 hours before toxin challenge, **(D)** mRNA treatment 48 hours before toxin challenge.

All control mice died within 24 hours after toxin injection, while the treated mice showed variable survival. Among the mRNA groups the linear vector showed 100% protection at all time points. The circular vector demonstrated 100% protection at 16- and 24-hour time points, but only 50% protection at 48-hour timepoint. No protection was observed at the 4-hour time point, due to low expression levels.

## 3 Discussion

### 3.1 Luciferase activity

Linear and circular vectors exhibit different expression patterns both *in vivo* an *in vitro*. During *in vitro* experiment linear vectors reached their maximum at the 16^th^ hour post-transfection, followed by a plateau lasting for about 5 hours and a subsequent dramatic decrease. Circular vectors reached their maximum at the 20^th^ hour post-transfection followed by a plateau lasting nearly 15 hours and then a gradual decrease in luciferase activity.

Surprisingly, the decrease in luciferase activity of the linear vectors is more pronounced *in vivo* compared to *in vitro* study. This may be explained by more complex influence of mice immune response to the vectors and requires additional studying. Common results were obtained by Wesselhoeft et al. They demonstrated more dramatic distinction between vectors’ expression *in vivo* in contrast to cell-culture experiment (18).

Among the linear vectors mRNA constructs capped with CleanCap generated the higher luciferase activity compared to ARCA-capped mRNA. Our results correlates with the work of McCaffrey et al. However, in our experiment the difference between the two capping systems is not so dramatic. This may be explained by lower capping efficacy of ARCA reagent, as it was not mentioned weather only capped mRNA was used in their experiment or total IVT products (19).

Among circular mRNA, vector bearing the HRV-B6 IRES exhibited approximately two-folds higher luciferase activity in comparison with the circular vector with CVB3 IRES. This data correlates with Chen et al. who showed that there are more effective IRESes than commonly used IRES of CVB3 (16). At the same time our results are controversial to the work of Unti and Jaffrey, who showed the superiority of CVB3 IRES against HRV B3 IRES in the Tornado system (20).

All in all, during the first 24 hours the linear vectors containing N1-methylpseudouridine outperformed the circular ones; however, by 48 hours the circular vectors surpassed the linear ones.

Also, *in vitro* luciferase assay revealed that linear mRNA containing canonical nucleotides produced low luciferase expression, whereas linear vectors, containing N1-methylpseudouridine demonstrated markedly higher levels of luciferase activity. This finding correlates with the work of Kariko et al. who demonstrated the significance of mRNA modification for increased stability (21).

At the same time the recent research on the N1-methylpseudouridine modification of mRNA causes concerns. Mulroney et al. showed the ribosome frame-shift caused by this modification. Along with *in vitro* detection of side peptides after transfection with this modified mRNA they found antibodies in mice to these peptides. This finding rises a problem of undesired immune response to non-target proteins and possible autoimmunity (22).

In summary, our luciferase experiments demonstrate that circular vectors exhibit a longer lasting expression in comparison with linear vectors, although their peak expression levels are lower.

### 3.2 SARS-Cov2 expression and immunization

In accordance with *in vitro* luciferase assay linear vectors with canonical nucleotides showed low, levels of protein expression (114 ng/mL for ARCA-U-S and 153 ng/mL for CleanCap-U-S).

The linear vector, modified with N1-methylpseudouridine and capped with CleanCap remained the highest performer as before. However, the linear vector capped with ARCA was displaced from second place by the circular vector with HRVB6 IRES (519 ng/mL and 875 ng/mL, respectively).

Our results correlate with findings by Qu L. et. al., who also demonstrated the superiority of circular mRNA over linear mRNA capped with cap0, however different IRESes were used (15).

The disparity between linear and circular vectors was reduced compared to the luciferase assay. This finding could be explained by the influence of the vectors` secondary structure, obtained by the combination of UTRs, introns, IRES and the gene. These interactions requires further research.

Immunogenicity study at a dose 10 µg of LNP-RNA per mouse demonstrated the decrease in the differences between the vectors, combining to luciferase assay. So that, circular vector with HRV B6 IRES showed immunogenicity, equal to that of the linear mRNA with CleanCap and significantly surpasses mRNA-vector with ARCA and circular vector with CVB B3 IRES.

Further decrease of immunization dose to 3 µg/mouse exhibited similar indicators for circular vector with HRV B6 IRES and the linear mRNA with CleanCap (end-point GMT, Viral load and survival).

Our data is consistent with the work of Qu L. et al. They showed that immunization at a dose of 2.5 µg/mouse provides equal endpoint antibody titres for linear and circular mRNA vectors. In contrast to our study, they performed a virus neutralization test instead of SARS-CoV2 challenge in mice, so it is not possible to completely compare protective activity. Nevertheless, both studies demonstrate in different ways that circular mRNA vaccine is an effective way to protect mice from SARS-CoV2. The distinction lies in the antibody titres for linear and circular mRNA in our work and theirs relates to the differences in the antigen used: we employed the gene of the hole Spike protein instead of RBD domain (15).

It is important to mention the size and structure of our poly-(A) tail. Due to the common problem of poly-(A) deletion in linear mRNA-templates during cultivation we designed an “island” containing 10 other nucleotides, resulting in a poly-(A) tail with a 30/70 ratio as opposed to 100-A poly-(A) used, by Qu L. et al. which can be deleted during cultivation causing lower results for the linear vector (15).

This finding demonstrates that as a vaccine platform the circular vector with IRES HRVB6 is not only superior to the linear mRNA capped with ARCA reagent but is also equivalent to the widely used mRNA capped with CleanCap and modified with N-1-Me-Pseudouridine. Also, this result is an evidence that immune response is a complex process, depending not only from expression level, but on a variety of factors (18).

### 3.3 Passive immunization

First of all, it is important to mention that the data on the use of circular mRNA for passive immunization is limited. At the same time, it was shown by the number of works, that linear mRNA is a suitable platform for passive immunization that was successfully used against viral pathogens, bacterial toxins, snake venom and different types of cancer (23–26).

So, the aim of this experiment was to evaluate the applicability of circular mRNA-vector, coding anti-toxin antibody, for prophylaxis of lethal outcome in mice after BoNT/A challenge.

*In vivo* kinetics of antibody expression showed the expected dramatic superiority of the linear vector over the circular one in the first 24 hours post-treatment. By the 48^th^ hour post-treatment, however, serum antibody concentrations were similar between the two groups.

Unfortunately, the antibody titre produced by the circular vector was near the detection limit at some timepoints, while the linear vector produced a more complete curve. We assume that the linear vector, consistent with luciferase kinetics, produces significant amounts of antibodies in the first hours, followed by a sequential decrease, whereas, the circular mRNA-vector continuously produces lower quantities of antibodies.

In our next experiment mice were challenge with 5LD_50_ of BoNT/A. The circular mRNA vector did not reach protective antibody concentrations within the first four hours post-treatment. At 16 hours, however, all mice had reached protective antibody concentrations. This effect was observed at the 24-hour time point. At 48-hour point, the survival in circular mRNA group was 50%, while the linear group had 100% survival. This data correlates with the luciferase assay, where 48-hour activity was approximately 75% of the 24-hour luciferase activity, corresponding to 75% protection from 5LD_50_.

Obtained results demonstrated that our circular mRNA vector is suitable for passive immunization, but with less efficacy than the linear one. For more effective use further improvements of expression efficacy, is needed or the dose increase and safety evaluation.

This reduced efficacy of circular vectors could be attributed, to immune response activation by the presence of impurities. Wesselhoeft RA et all demonstrated, that RNase R treatment alone does not prevent the activation of RIG-I and MDA-5 by circular RNA, while HPLC-purified RNA avoids this activation (18). Another approach for expression increase is the use of modified nucleotides analogues. At the same time some of the modified nucleotides causes absolute loss of expression of circular mRNA (18, 25, 27). Nevertheless, our research provided the first data on the use of circular mRNA-vectors for passive immunization.

At the same time the activation of native immune response in some cases may serves as a self-adjuvant increasing target immune response. On the one hand immune response activation decreases expression of the antigen, on the other hand it interacts cells, increasing the vaccine efficacy. The rational balance between these aspects should be provided by rational vector design (28–30).

## 4 Conclusion

Our work provides a comprehensive comparative analysis of different types of linear and circular mRNA vectors. Linear vectors were produced using either N-1-Me-Pseudouridine or Uridine and two capping reagents: ARCA or CleanCap. Circular vectors contained IRESes elements from Coxackie virus B3 or a novel human Rhinovirus B6.

We demonstrated that the luciferase assay should be considered as a preliminary tool, but should not serve as the sole criterion for comparing vaccine vectors Likewise, expression or immunogenicity studies alone are insufficient. All these assays should be used in combination with protective activity studies for a comprehensive evaluation. Additionally, we obtained a circular vector that provides immunogenicity and protective activity equal to that of the linear vector with CleanCap. Our results provide further evidence that circular mRNA is a relatively new and powerful tool for vaccine development and production, capable of competing with widely used linear mRNAs.

At the same time the data on the “frameshifting expression” of side proteins by linear vectors containing N1-methylpseudouridine are causing concerns on their safety, making circular RNA more appropriate platform for vaccine and theraupethic production.

## 5 Materials and methods

### 5.1 Cells and bacterial strains and viruses

*Escherichia coli* DH10B (Invitrogen, USA) was used for DNA preparation. HEK-293 and Vero E6 cells were purchased from ATCC (CRL-1573 and CRL-1586. respectively). Cells were cultured at 37°C, in a humidified incubator with 5% atmosphere of CO_2_. Dulbecco`s Modified Eagle Medium (Corning, 10-013-CV) was used for cultivation. HEK-293 medium contained 10% heat inactivated fetal bovine serum (HI-FBS), while Vero cell medium contained 2% HI-FBS.

### 5.2 mRNA production

DNA-templates for mRNA-production were obtained by Gibson cloning methods using GeneArt™ Gibson Assembly HiFi Master Mix (Invitrogen™, A46628) according to the manufacturer`s instructions. Correct assembly was confirmed via Sanger sequencing. Purified DNA-templates were linearized overnight and complete digestion was confirmed with agarose gel electrophoresis.

mRNA was produced via *in vitro* transcription (IVT) using the HiScribe T7 High Yield mRNA Synthesis Kit (NEB #E2040S) according to the manufacturer`s recommendations. The reaction mix for circular mRNA contained canonical nucleotides and no Cap analogues, while the linear production mix was prepared in different variations: it contained either Uridine-5-Triphosphate or 1-Methylpseudouridine-5-Triphosphate (YEASEN # 10657ES20) and one of two capping reagents the m^7^G(5′)ppp(5′)m^2^G mRNA cap structure analogue (TriLink^®^#Т7113) or the anti-reverse cap analogue (Thermo, #AM8045).

The next step for both mRNA-types was treatment with DNase I (New England Biolabs #M0303S) for 30 minutes at 37 °C to digest the DNA templates.

For circular mRNA there was an additional cyclization step. GTP was added to the linear precursor to a final concentration of 2 mM and incubated at 55 °C for 15 minutes to catalyze the cyclization reaction. Then circular mRNA was purified with the Monarch mRNA Cleanup Kit (New England Biolabs #T2040L). To degrade residual linear precursor, samples were heated at 65 °C for 3 minutes and cooled on ice before RNase R treatment (Epicenter #RNR07250) at 37 °C for 30 minutes.

Linear mRNA was purified with the Monarch mRNA Cleanup Kit (New England Biolabs #T2040L). Then uncapped and nicked RNA was removed via enzymatic degradation with XRN-1 (NEB #M0338) and RppH (NEB #M0356) at 37 °C for 1 hour.

Final IVT products were purified with the mRNA Clean & Concentrator Kit (ZYMO #R1018).

### 5.3 *In vitro* firefly luciferase assay

HEK-293 cells were seeded into white FB/HB 96-well plates (Greiner) at 2 x 10^4^ per well in 150 µl of DMEM. Six hours later, after cells had finally attached to the bottom, they were transfected with various luciferase-coding mRNAs using with Lipofectamine™ 2000 Transfection Reagent (Invitrogen™) according to the manufacturer’s instructions. The dose of mRNA was 20 ng per well. Fifteen minutes after transfection, D-luciferin (Promega) was added to a final concentration of 5 mM.

Measurement of *in vitro* luciferase kinetics was performed using a CLARIOstar plate reader (BMG Labtech) equipped with an Atmospheric Control Unit. Cells were cultured in 5% CO_2._ at 37 °C. Luciferase activity was measured every hour. All the experiments were performed in triplicate with different IVT-batches and cell passages.

Transfection was performed using Lipofectamine™ 2000 Transfection Reagent (Invitrogen™) as per the manufacturer’s instructions.

### 5.4 *In vitro* expression of SARS-Cov2

HEK-293 cells were seeded to 6-well plates at 3 x 10^5^ cells per well. The next day, when confluency reached 70% cells were transfected with different Spike-coding mRNAs at a dose of 1 µg per well using Lipofectamine™ 2000 Transfection Reagent (Invitrogen™) according to the manufacturer’s instructions. Twenty hours post-transfection cells were collected, washed three times with PBS and lysed. Cell lysates were centrifuged and used for ELISA assay.

The day prior to the analysis, 1p1B10-Fc antibodies were adsorbed onto 96-well plates (Costar, USA), at +4 °C overnight (31). The quantity of antibodies was 100 ng per well. Then the plate was washed three times with 0.05% Tween-20 PBS (TPBS). After one-hour of blocking and a triple wash with TPBS, cell lysate supernatant and control samples were added. Recombinant S-glycoprotein of SARS-CoV2-Wuhan (Sino Biological, China) was used as a positive control. After one-hour of incubation at 37 °C and five washes with TPBS wash detecting antibodies were added.

As primary antibodies we used 1:1000 diluted polyclonal serum from mice immunized with S-glycoprotein of SARS-CoV2. Detection was performed at 37 °C for one hour followed by five washes with TPBS.

Secondary antibodies were then added with additional one-hour incubation and five TPBS washes. As secondary antibodies we used HPR-conjugated sheep anti-mouse antibodies (NA931V, GE Healthcare, USA).

Finally, TMB substrate (Immunotech, Russia) was added and incubated for 30 minutes at 37 °C. The reaction was stopped by addition of 1М H_2_SO_4_. OD_450_ was measured using a CLARIOstar plate reader (BMG Labtech).

### 5.5 Formulation of mRNA in lipid nanoparticles

Lipid nanoparticles (LNP) assembly procedure was performed as previously described (32). briefly, lipid nanoparticle included the components, listed below: ionizable lipid (ALC-0315), DSPC, cholesterol and PEG-lipid. Their molar ratio was 46.3:9.4:42.7:1.6. Lipid components were dissolved in ethanol, while purified mRNA was dissolved in 10 mM sodium citrate buffer (pH 3.0) to a final concentration 0.2 mg/mL. Then aqueous and ethanol fractions were mixed in ration 3:1 using a Nanoassemblr Spark device (Precision NanoSystems, USA). Obtained substance was dialyzed against PBS (pH 7.2) with 10% sucrose in Slide-A-Lyzer dialysis cassettes (Thermo Fisher Scientific, USA) for 24 hours. Then formulation was filter sterilized through 0.22 μm Briefly, the lipid nanoparticle formulation included the components, listed below: ionizable lipid (ALC-0315), DSPC, cholesterol and PEG-lipid in a molar ratio of 46.3:9.4:42.7:1.6.

Lipid components were dissolved in ethanol, while purified mRNA was dissolved in 10 mM sodium citrate buffer (pH 3.0) to a final concentration of 0.2 mg/mL. The aqueous and ethanol fractions were mixed in a ratio of 3:1 using a Nanoassemblr Spark device (Precision NanoSystems, USA). Obtained substance was dialyzed against PBS (pH 7.2) with 10% sucrose in Slide-A-Lyzer dialysis cassettes (Thermo Fisher Scientific, USA) for 24 hours.

Then formulation was filter-sterilized through 0.22 μm PES-filter syringe. Prior to use, lipid nanoparticle characteristics were measured: diameter, size distribution and Zeta-potential were evaluated using Zetasizer Nano ZS instrument (Malvern Panalytical) according to the manufacturer`s manual. Total encapsulation efficiency and concentration of mRNA in final formulations were determined by RiboGreen assay (Quant-iT™ RiboGreen™ mRNA Reagent, Thermo Fisher Scientific) as previously described (32).

### 5.6 SARS-CoV-2 Preparation

The Wuhan-like SARS-CoV-2 virus strain hCoV-19/Russia/Moscow_PMVL-1/2020 was used in the study. SARS-CoV-2 virus was propagated in Vero E6 cells in DMEM with 2% HI-FBS, harvested after 72 hours, aliquoted, titrated on Vero E6 cells and stored at −80 °C. The virus titer was determined on Vero E6 cells using a 50% tissue culture infectious dose (TCID_50_) assay. Serial 10-fold dilutions of the virus stock were prepared in DMEM with 2% HI-FBS and in the volume of 100 μL were added to Vero E6 cells in a 96-well plate in 8 replicates. The cells were incubated at 37 °C in 5% CO_2_ for 96–120 hours and scored visually for cytopathic effect. The TCID_50_ titer was calculated by the Spearman–Kerber method.

### 5.7 Ethical statement

All animal experiments were approved by the Institutional Animal Care and Use Committee (IACUC) of the Federal Research Centre of Epidemiology and Microbiology named after Honorary Academician N.F. Gamaleya (protocol #93 of May 14, 2025). All procedures with SARS-CoV-2 were carried out in approved biosafety level 3 facilities. All animal experiments were performed in strict accordance with the recommendations of the National Standard of the Russian Federation (33).

### 5.8 Animal experiments

Protective efficacy was studied in 6–7 week old hemizygous K18-ACE2-transgenic F1 mice obtained by crossing transgenic males B6.Cg-Tg(K18-ACE2)2Prlmn/J, health status SOPF (Jackson Laboratory, USA) and non-transgenic females C57BL/6 Gamrc health status SPF (Gamaleya Research Center, Russia). BALB/c mice were used for *in vivo* luciferase assay and for B11-Fc study. Mice had free access to water and food and were housed in an ISOcage N system (Tecniplast, Buguggiate, Italy).

For the *in vivo* luciferase assay mice were randomly divided into five groups and were treated intramuscularly with LNPs, containing different luciferase-coding mRNAs at a dose of 10 µg per mouse. At 10, 24, 48 and 72 hours post-mRNA treatment, 2.5 µg of D-luciferin was injected intraperitoneally. For SARS-CoV2 studies, mice were immunized twice with S-glycoprotein coding mRNA-vectors at a dose of 3 or once at a dose of 10 µg. As a negative control, luciferase-coding linear mRNA capped with CleanCap reagent and containing N1-methylpseudouridine was used. The interval between immunizations was 21 days. Blood was collected from five mice of each group 21 days after the first of the second immunization using local anesthesia with lidocaine. The size of blood probe did not exceed 100 µl per mouse.

The remaining mice in each group were challenged intranasally at a dose of 10^5^ TCID_50_ with SARS-CoV-2 virus (Wuhan-like). Five animals per group were euthanized on day 4 post-challenge for macroscopic analysis of lung damage and determination of viral load in the lungs. Euthanasia was performed using an overdose of injectable anesthesia. After autopsy the lungs were removed and washed with PBS. A piece of tissue was dissected, placed in homogenization tubes, weighed and supplemented with PBS at 90% of the tissue weight.

Another ten animals per group were monitored for weight loss and survival for 21 days. Animals that lost more than 20% of their initial body weight were euthanized before the end of the study.

For the therapeutic potential study of mRNA, mice were treated with 10 µg of B11-Fc-coding mRNA; the negative control remained the same. Then blood was collected at 8, 24, 48 and 72 hours post-injection (three mice per group, per time-point). The procedure for blood collection is described above.

Botulinum toxin A was obtained as previously described (34). The toxin was injected intraperitoneally at a dose of 5LD_50_ at different time-points after mRNA-treatment. Mice were observed for 48 hours. Death time was defined as the time when a mouse was found dead or was euthanized at a humane endpoint by carbon dioxide asphyxiation followed by cervical dislocation.

### 5.9 *In vivo* kinetics of B11-Fc expression by linear and circular mRNA vectors

The concentration of recombinant B11-Fc antibody was measured using ELISA kit IgG total-EIA-BEST (Vector-Best, Russia), according to the manufacturer`s instructions, with the following modifications: substitution of kit`s secondary antibodies with ECL Human IgG, HRP-linked whole Ab (Cytiva, NA933-1ML) and use of purified B11-Fc instead of kit`s standard sample.

### 5.10 Endpoint anti-Spike antibodies GMT measurement

An ELISA protocol was used to evaluate anti-RBD antibodies. Serum samples were purified by centrifugation (800× g, 10 min). Recombinant SARS-CoV-2 RBD domain of B.1.1.1 SARS-CoV-2 virus (SinoBiological, Beijing, China) was used for overnight coating of 96-well plates (100 ng/well). Plates were washed five times with washing solution (PBS + 0.1% Tween-20. TPBS) and then blocked with blocking solution (TPBS with 5% non-fat dry milk). Serum samples were serially diluted in blocking solution and added to wells, followed by incubation at 37 °C for 1 hour. After washing, anti-mouse total IgG secondary HRP-conjugated antibodies (1:5000 dilution, Abcam, Cambridge, UK) in blocking solution were added and plates were incubated at 37 °C for 1 hour. After a final wash, TMB substrate was added and plates were incubated at 20–25°С. The reaction was stopped with 4 M H_2_SO_4_. The colorimetric signal was measured at 450 nm using a Multiscan FC spectrophotometric plate reader (Thermo Fisher Scientific, Waltham, MA, USA) 30 minutes after the addition of stop solution. The ELISA titer was defined as the highest serum dilution, that produced a colorimetric signal at least twice that of the corresponding dilution of the control serum.

### 5.11 Determination of viral load in lungs

Ten percent lung homogenates in DMEM with 2% heat-inactivated FBS were prepared on day 4 after challenge using an MPbio FastPrep-24 (MP Biomedicals, Irvine, CA, USA). Homogenates were centrifuged at 12.000× g for 10 minutes and the supernatant were used for further analysis.

The infectious virus titer was determined as described above on Vero E6 cells in a 96-well plate in four replicates after a 120-hour incubation.

mRNA was isolated from virus-containing samples using RNeasy Plus Universal Kits (QIAGEN, Germany) according to the manufacturer’s protocol. RV-RT-PCR was performed using the POLIVIR SARS-CoV-2 “Express” kit (Lytech, Russia) as per manufacturer’s instructions. SARS-CoV-2 viral mRNA with a known concentration of GE/ml was used to construct the calibration curve.

### 5.12 Statistical analysis and graphical design

All statistical calculations were performed using GraphPad Prism 10. The normality of data distribution was evaluated in the d’Agostino–Pearson test. Comparisons of unpaired samples were performed by the Mann–Whitney test. Multiple data sets were compared by non-parametric ANOVA (Friedman’s test) with Dunn’s multiple comparison post-test. Survival curves were compared using the Log-rank (Mantel–Cox) test.

Visual schemes of the experiments were made using Biorender.

## Data Availability

The datasets presented in this study can be found in online repositories. The names of the repository/repositories and accession number(s) can be found below: https://www.ncbi.nlm.nih.gov/genbank/, DQ473486.1 https://www.ncbi.nlm.nih.gov/genbank/, M33854.1 https://www.ncbi.nlm.nih.gov/genbank/, OR134577.1.
