# Supplementary material for: Circular mRNA against CleanCap linear mRNA vectors: comprehensive comparison, expression, active and passive immunization
Source: Front Immunol. 2026 Apr 24;17:1734751. doi: 10.3389/fimmu.2026.1734751 (PMC13153118; doi:10.3389/fimmu.2026.1734751)
Supplement: Supplementary file 1 [file DataSheet1.pdf]

Supplementary Material

1 Supplementary Data

2 Supplementary Figures and Tables

2.1 Supplementary Figures

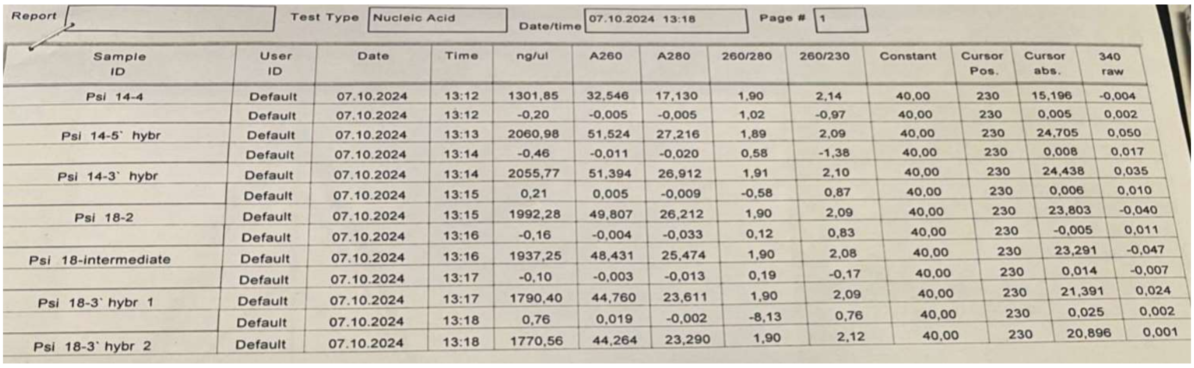

Report | Test Type: Nucleic Acid | Date/time: 07.10.2024 13:18 | Page #: 1

Sample ID	User ID	Date	Time	ng/ul	A260	A280	260/280	260/230	Constant	Cursor Pos.	Cursor abs.	340 raw
Psi 14-4	Default	07.10.2024	13:12	1301,85	32,546	17,130	1,90	2,14	40,00	230	15,196	-0,004
	Default	07.10.2024	13:12	-0,20	-0,005	-0,005	1,02	-0,97	40,00	230	0,005	0,002
Psi 14-5` hybr	Default	07.10.2024	13:13	2060,98	51,524	27,216	1,89	2,09	40,00	230	24,705	0,050
	Default	07.10.2024	13:14	-0,46	-0,011	-0,020	0,58	-1,38	40,00	230	0,008	0,017
Psi 14-3` hybr	Default	07.10.2024	13:14	2055,77	51,394	26,912	1,91	2,10	40,00	230	24,438	0,035
	Default	07.10.2024	13:15	0,21	0,005	-0,009	-0,58	0,87	40,00	230	0,006	0,010
Psi 18-2	Default	07.10.2024	13:15	1992,28	49,807	26,212	1,90	2,09	40,00	230	23,803	-0,040
	Default	07.10.2024	13:16	-0,16	-0,004	-0,033	0,12	0,83	40,00	230	-0,005	0,011
Psi 18-intermediate	Default	07.10.2024	13:16	1937,25	48,431	25,474	1,90	2,08	40,00	230	23,291	-0,047
	Default	07.10.2024	13:17	-0,10	-0,003	-0,013	0,19	-0,17	40,00	230	0,014	-0,007
Psi 18-3` hybr 1	Default	07.10.2024	13:17	1790,40	44,760	23,611	1,90	2,09	40,00	230	21,391	0,024
	Default	07.10.2024	13:18	0,76	0,019	-0,002	-8,13	0,76	40,00	230	0,025	0,002
Psi 18-3` hybr 2	Default	07.10.2024	13:18	1770,56	44,264	23,290	1,90	2,12	40,00	230	20,896	0,001

Supplementary Figure 1. Results of absorbance and concentration measurement linear vectors, containing N1-Me-Ψ; 14-4 – CleanCap-Luc, 14-5` – ARCA-Luc, 18-2 – CleanCap-S, 18-Intermediate – ARCA-S.

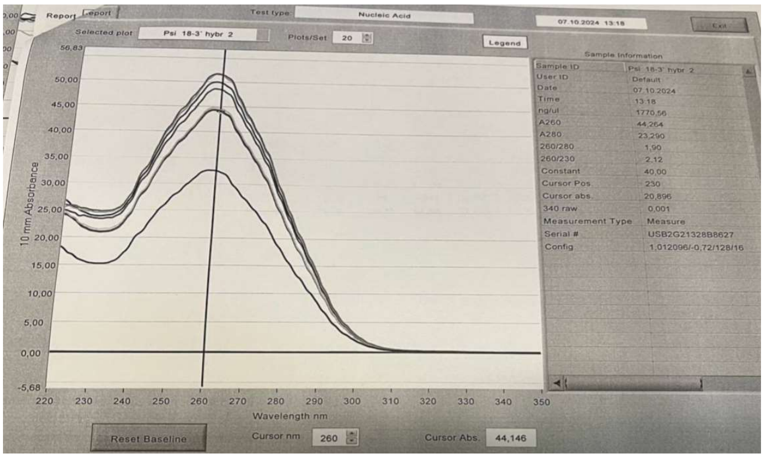

Supplementary Figure 2. Absorbance curve of linear vectors, containing N1-Me-Ψ; 14-4 – CleanCap-Luc, 14-5` – ARCA-Luc, 18-2 – CleanCap-S, 18-Intermediate – ARCA-S.

Report | Test Type: Nucleic Acid | Date/time: 07.10.2024 13:06 | Page # 1

Sample ID	User ID	Date	Time	ng/ul	A260	A280	260/280	260/230	Constant	Cursor Pos.	Cursor abs.	340 raw
U 14-4	Default	07.10.2024	12:58	2231,77	55,794	27,773	2,01	2,13	40,00	230	26,234	-0,080
	Default	07.10.2024	12:58	1,80	0,045	0,041	1,10	3,89	40,00	230	0,012	0,024
U 14-5' hybr	Default	07.10.2024	12:58	2289,97	57,249	26,968	2,12	2,27	40,00	230	25,214	-0,202
	Default	07.10.2024	12:59	44,29	1,107	0,681	1,63	1,16	40,00	230	0,958	0,116
	Default	07.10.2024	12:59	77,76	1,944	1,187	1,64	1,16	40,00	230	1,682	0,233
	Default	07.10.2024	13:00	-0,25	-0,006	-0,002	2,54	0,39	40,00	230	-0,016	-0,005
U 14-3' hybr	Default	07.10.2024	13:00	2331,76	58,294	27,517	2,12	2,23	40,00	230	26,116	-0,058
	Default	07.10.2024	13:01	0,57	0,014	0,023	0,61	-15,69	40,00	230	-0,001	-0,014
U 18-2'	Default	07.10.2024	13:02	2291,36	57,284	26,996	2,12	2,26	40,00	230	25,321	-0,216
	Default	07.10.2024	13:02	-0,32	-0,008	0,005	-1,65	1,13	40,00	230	-0,007	0,002
U 18-Intermediate	Default	07.10.2024	13:03	1765,93	44,148	20,622	2,14	2,27	40,00	230	19,406	-0,297
	Default	07.10.2024	13:05	0,07	0,002	0,002	0,73	-0,11	40,00	230	-0,016	0,012
U 18-3' hybr1	Default	07.10.2024	13:05	1958,68	48,967	23,045	2,12	2,28	40,00	230	21,499	-0,203
	Default	07.10.2024	13:06	2,07	0,052	0,039	1,32	1,70	40,00	230	0,030	0,003
U 18-3' hybr2	Default	07.10.2024	13:06	2006,42	50,161	23,518	2,13	2,27	40,00	230	22,110	-0,189

Supplementary Figure 3. Results of absorbance and concentration measurement linear vectors, containing U; 14-4 – CleanCap-Luc, 14-5` – ARCA-Luc, 18-2 – CleanCap-S, 18-Intermediate – ARCA-S.

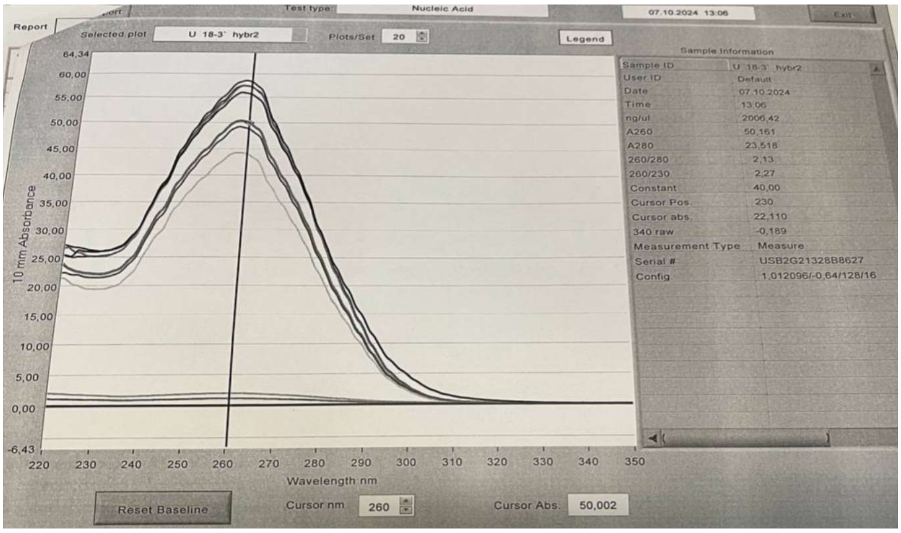

Supplementary Figure 4. Absorbance curve of linear vectors, containing N1-Me-Ψ; 14-4 – CleanCap-Luc, 14-5` – ARCA-Luc, 18-2 – CleanCap-S, 18-Intermediate – ARCA-S.

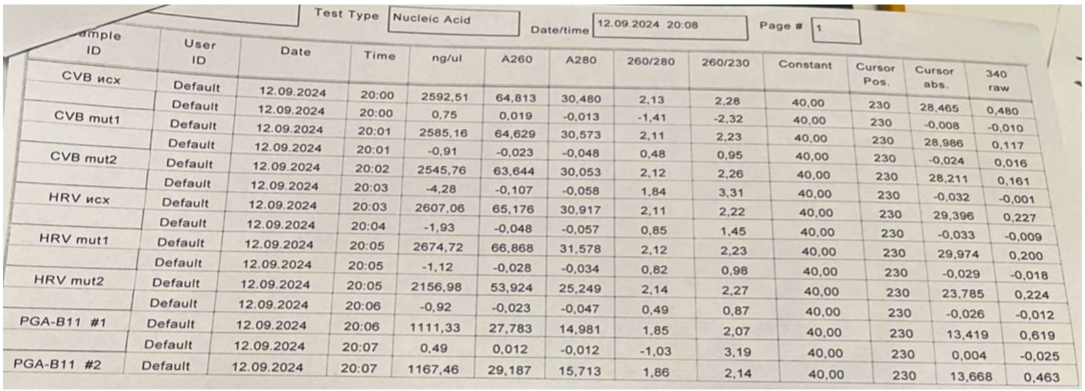

Test Type: Nucleic Acid | Date/time: 12.09.2024 20:08 | Page # 1

Sample ID	User ID	Date	Time	ng/ul	A260	A280	260/280	260/230	Constant	Cursor Pos.	Cursor abs.	340 raw
CVB исх	Default	12.09.2024	20:00	2592,51	64,813	30,480	2,13	2,28	40,00	230	28,465	0,480
	Default	12.09.2024	20:00	0,75	0,019	-0,013	-1,41	-2,32	40,00	230	-0,008	-0,010
CVB mut1	Default	12.09.2024	20:01	2585,16	64,629	30,573	2,11	2,23	40,00	230	28,986	0,117
	Default	12.09.2024	20:01	-0,91	-0,023	-0,048	0,48	0,95	40,00	230	-0,024	0,016
CVB mut2	Default	12.09.2024	20:02	2545,76	63,644	30,053	2,12	2,26	40,00	230	28,211	0,161
	Default	12.09.2024	20:03	-4,28	-0,107	-0,058	1,84	3,31	40,00	230	-0,032	-0,001
HRV исх	Default	12.09.2024	20:03	2607,06	65,176	30,917	2,11	2,22	40,00	230	29,396	0,227
	Default	12.09.2024	20:04	-1,93	-0,048	-0,057	0,85	1,45	40,00	230	-0,033	-0,009
HRV mut1	Default	12.09.2024	20:05	2674,72	66,868	31,578	2,12	2,23	40,00	230	29,974	0,200
	Default	12.09.2024	20:05	-1,12	-0,028	-0,034	0,82	0,98	40,00	230	-0,029	-0,018
HRV mut2	Default	12.09.2024	20:05	2156,98	53,924	25,249	2,14	2,27	40,00	230	23,785	0,224
	Default	12.09.2024	20:06	-0,92	-0,023	-0,047	0,49	0,87	40,00	230	-0,026	-0,012
PGA-B11 #1	Default	12.09.2024	20:06	1111,33	27,783	14,981	1,85	2,07	40,00	230	13,419	0,619
	Default	12.09.2024	20:07	0,49	0,012	-0,012	-1,03	3,19	40,00	230	0,004	-0,025
PGA-B11 #2	Default	12.09.2024	20:07	1167,46	29,187	15,713	1,86	2,14	40,00	230	13,668	0,463

Supplementary Figure 5. Results of absorbance and concentration measurement of circular vectors and linear vector, coding B11-antibody. CVBисх – Luc-coding, CVB mut1 – S-glycoprotein coding, HRVисх – Luc-coding, HRV mut1 – S-glycoprotein coding, HRV mut2 – B11-coding. PGA-B11 #1,2 – two clones of Linear CleanCap-B11, containing N1-Me-Ψ.

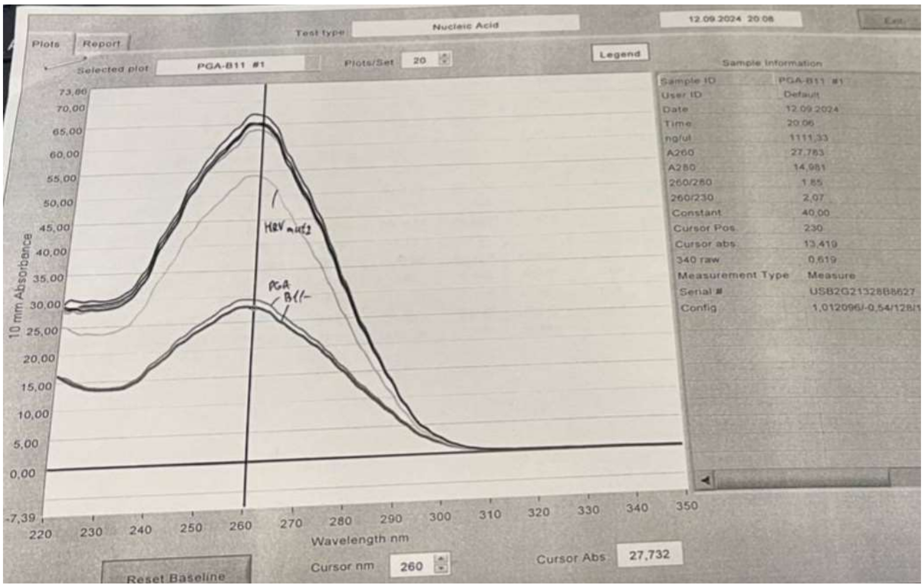

Supplementary Figure 6. Absorbance curve of circular vectors and linear vector, coding B11-antibody.

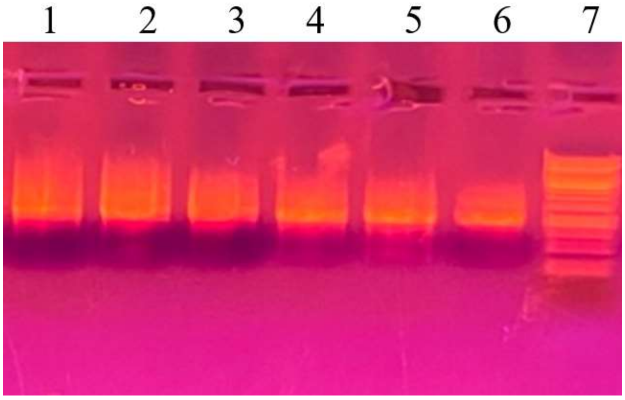

Supplementary Figure 7. Gel-electrophoresis of Firefly Luciferase-coding RNA in 1% agarose gel. 1 – Circular mRNA, coding Firefly luciferase containing IRES of HRVB6, 2 – Circular mRNA, coding Firefly luciferase containing IRES of CVB3, 3 – Linear mRNA, coding Firefly luciferase capped with ARCA-reagent, containing N1-Me-Ψ, 4 – Linear mRNA, coding Firefly luciferase capped with ARCA-reagent, containing U, 5 – Linear mRNA, coding Firefly luciferase capped with CleanCap-reagent, containing U, 6 – Linear mRNA, coding Firefly luciferase capped with CleanCap-reagent, containing N1-Me-Ψ, 7 – Gene ruler 1 kb DNA ladder

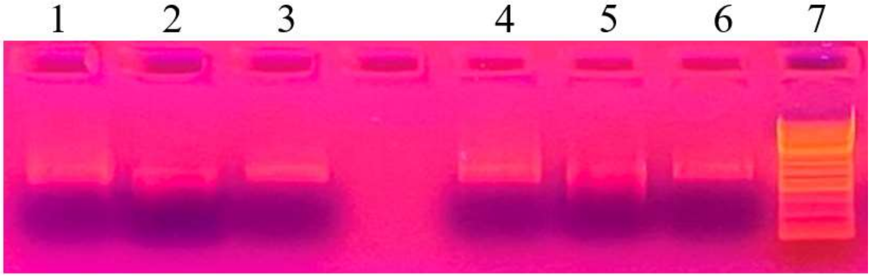

Supplementary Figure 8. Gel-electrophoresis of S-glycoprotein-coding RNA in 1% agarose gel. 1 – Linear mRNA, coding S-glycoprotein capped with ARCA-reagent, containing U; 2 – Linear mRNA, coding S-glycoprotein capped with CleanCap-reagent, containing U; 3 – Circular mRNA, coding S-glycoprotein containing IRES of CVB3; 4 – Circular mRNA, coding S-glycoprotein containing IRES of HRVB6; 5 – Linear mRNA, coding S-glycoprotein capped with ARCA-reagent, containing N1-Me-Ψ; 6 – Linear mRNA, coding S-glycoprotein capped with CleanCap-reagent, containing N1-Me-Ψ; 7 – Gene ruler 1 kb DNA ladder

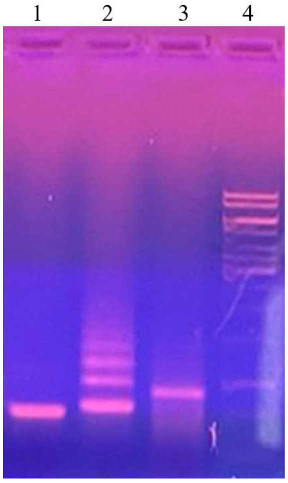

Supplementary Figure 9. Gel-electrophoresis of B11-coding RNA in 1% agarose gel. 1 – Linear mRNA, coding B11-antibody capped with CleanCap-reagent containing N1-Me-Ψ; 2 – Linear mRNA, coding Firefly luciferase, obtained from positive control DNA-template in HiScribe T7 Quick High Yield RNA; 3 – Circular mRNA, coding B11-antibody containing IRES of HRVB6; 4 – Gene ruler 1 kb DNA ladder.
